# Non-dominant leg joints bear greater loading during balance beam walking in 4-year-old children

**DOI:** 10.3389/fspor.2025.1660112

**Published:** 2025-10-03

**Authors:** Jintao Pan, Zihang Xu, Xue Hu, Weixin Zhu, Qining Yang, Xiping Ren

**Affiliations:** ^1^ College of Physical Education and Health Sciences, Zhejiang Normal University, Jinhua, China; ^2^ Department of Rehabilitation, The Affiliated Jinhua Hospital, Zhejiang University School of Medicine, Jinhua, China; ^3^ Department of Joint Surgery, The Affiliated Jinhua Hospital, Zhejiang University School of Medicine, Jinhua, China

**Keywords:** early childhood, narrow beam, functional tasks, dynamic balance, biomechanics

## Abstract

**Background:**

Dynamic balance is a critical foundation for the development of motor skills in early childhood. Functional tasks such as beam walking pose a significant challenge to the frontal plane stability of preschool children. However, the mechanisms by which young children regulate hip, knee, and ankle joint loading under such conditions remain unclear. Therefore, this study aimed to explore the regulatory strategies of lower limb joint reaction forces during beam walking in 4-year-old children.

**Methods:**

Fourteen healthy 4-year-old children participated in overground walking (OGW) and balance beam walking (BBW). A markerless motion capture system, OpenCap, was used to collect kinematic data. Joint reaction forces in the frontal plane for the dominant and non-dominant at the hip, knee, and ankle were computed using OpenSim. One-dimensional time series parameters of joint reaction forces were used to assess loading characteristics between OGW and BBW.

**Results:**

Under BBW, the medial reaction force at the non-dominant hip joint significantly increased during multiple phases of the gait cycle, and the lateral force at the non-dominant knee joint decreased during the swing phase, with slower medial-to-lateral transitions.

**Conclusion:**

In functional walking tasks, asymmetry in lower limb joint loading between the dominant and non-dominant legs may serve as a sensitive indicator for assessing the neuromuscular development and gait control strategies in preschool children.

## 1 Introduction

Balance is a core component of early motor skill development in children and plays a crucial role in maintaining gait stability, improving motor coordination, and preventing injuries (1, 2). As the neuromuscular system matures, children's balance control abilities continue to develop and are optimized. Especially in the preschool stage, the rate of development of balance control in children is significant (3, 4), yet dynamic balance control in walking exhibits great variability (5).

Among the functional tasks designed to assess dynamic balance, narrow beam walking increases the difficulty of walking and places higher demands on body stability control. Not only is this task applicable to assessing balance function (6, 7), but it can also be used to evaluate the effectiveness of balance training (8). Beam walking has been applied to screen motor skill development and early identification of disorders in children (9). Studies have shown that in children with vestibular dysfunction, motor coordination disorders, and intellectual disability, beam walking can be employed as a potential early warning tool to help clinicians detect abnormal tendencies in motor control before symptoms become apparent, thus enabling early intervention and treatment (10). Age 4 is considered to be a critical period for the transition from the initial establishment of gait stability to fine regulation (3). At this age, children's neuromuscular system is still developing, and they may exhibit great gait variability and regulation variations when faced with challenging tasks such as beam walking (11). Reducing walking speed, decreasing step frequency, and increasing lower limb joint range of motion can maintain balance. These compensatory mechanisms often lead to increased energy expenditure and changes in joint loading (12).

Relative to spatiotemporal parameters (e.g., step length and step frequency), center of mass trajectories, and limb kinematics and dynamics (13, 14), the distribution of mechanical loading at the hip, knee, and ankle joints during gait control in preschoolers has not been fully studied. Previous studies have shown that there are significant differences in gait asymmetry among children with different weights (15). Additionally, gait symmetry significantly affects joint loading in the frontal plane (16), potentially leading to asymmetric loading between the dominant and non-dominant legs (17). However, there is currently a lack of quantitative evidence to confirm whether such asymmetry affects joint loading in functional tasks requiring greater frontal stability.

Therefore, this study aimed to investigate the dynamic changes at the hip, knee, and ankle joint reaction forces in the frontal plane during balance beam walking (BBW) and overground walking (OGW) in 4-year-old children, and to reveal the loading regulation strategies of lower limb joints in complex functional balance tasks. We hypothesized that lower limb joint loading adjustments and balance control strategies during BBW in 4-year-old children may exhibit asymmetrical patterns.

## 2 Methods

### 2.1 Study design

An observational study.

### 2.2 Participants

The sample size was computed using G*Power software (v3.1.9.7, University of Düsseldorf, Germany), based on *a priori* power analysis assuming a difference between two dependent means (*α* = 0.05, 1-*β* = 0.80, effect size dz = 0.81) (18). Fourteen 4-year-old children from a local preschool participated in this study (Table 1). Regarding walking on a balance beam, all participants had no prior experience. The dominant leg of all participants was determined based on the leg used for kicking (19, 20), identified as the right leg. Inclusion criteria were the absence of neurological dysfunction, musculoskeletal disorders, and psychological disorders. Written consent was obtained from their parents prior to the measurements. Ethical approval was obtained from the Ethics Committee of Zhejiang Normal University (ZSRT2024203). All measurements were carried out in accordance with the Declaration of Helsinki.

**Table 1:** Demographic characteristics of preschool children in this study. Values are presented as mean ± standard deviation (SD).

N	Gender (M/F)	Age (years)	Height (m)	Body Mass (kg)	BMI (kg/m^2^)
14	8/6	4.57 ± 0.35	1.12 ± 0.05	17.82 ± 1.42	14.22 ± 0.74

### 2.3 Experimental protocol

**Figure 1:**
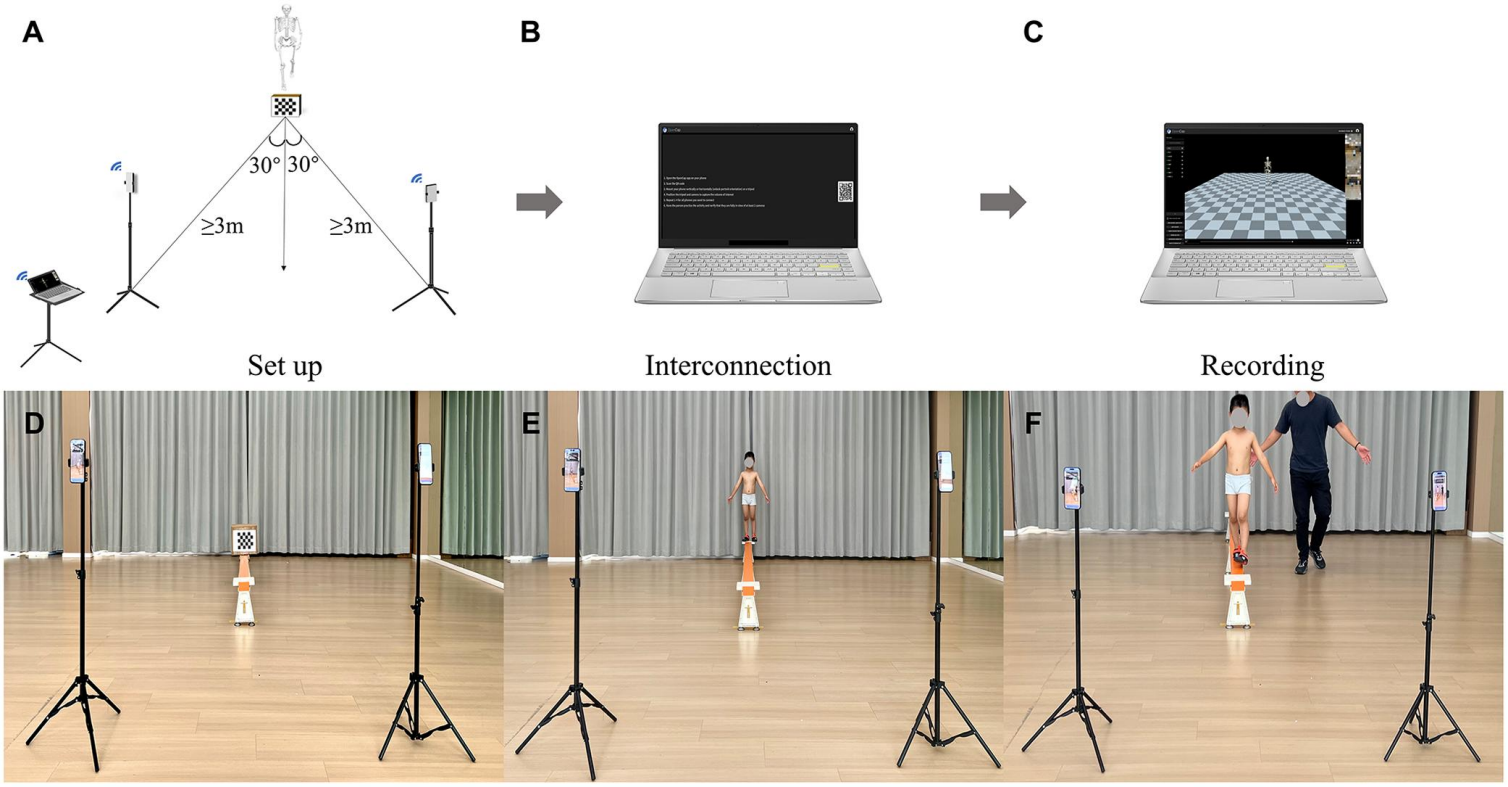
Schematic diagram of the experiment. The entire system consisted of three main components: scene setup **(A)**, online network connection **(B)**, and motion capture video recording **(C)** The apparatus included two iPhones mounted on tripods with phone holders, a 210 × 175 mm checkerboard (five rows, six columns, 35 mm square size) printed on A4 paper, and a laptop running the OpenCap system. Balance beam walking (BBW) data acquisition involves motion space calibration **(D)**, human neutral posture calibration **(E)**, and walking video recording **(F)** The overground walking (OGW) experimental process was the same as BBW, with the balance beam replaced by 3 m bright yellow tape on the floor.

A schematic diagram of the experimental setup, including the apparatus configuration, calibration space, and data acquisition workflow, is shown in Figure 1. A standard-sized balance beam measuring 3 m in length, 0.1 m in width, and 0.3 m in height (Reliable Co., Ltd., Beijing, China) served as an apparatus for the balance tasks. Each end of the beam was equipped with a 0.2 × 0.3 m elevated platform (height: 0.3 m) serving as a safe starting and ending area (21). Additionally, a straight line measuring 3 m in length and 0.1 m in width was marked on the ground using bright yellow tape, serving as the walking path. Two iOS devices (iPhone 13 Pro Max and iPhone XR, Apple Inc., Cupertino, CA, USA) integrated with an OpenCap mobile application (version 1.6, Model Health, Inc., Stanford University, USA) were mounted on adjustable tripods for motion capture, which has been confirmed to be sufficient for analyzing movements such as walking (22). The two iPhone cameras were positioned at approximately ±30° from the walking direction, at a minimum distance of 3 m from the movement space to reduce occlusion and ensure that the entire space was covered (22). These cameras operated at a frame rate of 60 Hz and a resolution of 720 × 1,280 pixels to record walking videos.

Camera calibration and motion space calibration were performed before data acquisition, following the steps outlined below. First, OpenCap automatically loaded intrinsic parameters of algorithms related to the principal point, focal length, and distortion parameters of the two camera hardware units for camera calibration. Subsequently, a printed 210 × 175 mm checkerboard (5 rows, 6 columns, 35 mm square size) on A4 paper taped to plexiglass perpendicular to the ground was placed in the view of two cameras for motion space calibration (22). The calibration checkerboard's accuracy has been validated (22). Next, both cameras captured the participant's still neutral pose. OpenCap scaled a musculoskeletal model to the child's anthropometry using OpenSim's Scale tool, based on the anatomical marker positions derived from the neutral pose.

Prior to data acquisition, children underwent a familiarization block with the OGW and BBW trials to familiarize themselves with the experimental environment. Data acquisition involved two blocks. First, children were asked to walk normally along a bright yellow line on the ground for 3 m. Then, they walked steadily from the starting point to the end of the beam. Both blocks require children to complete three trials at a comfortable pace employing a heel-to-toe walking pattern (23) and keeping arms naturally extended at their sides (24). A well-trained investigator closely monitored children throughout the process to prevent potential falls (24). If any child deviated from the marked line or showed any significant deviation at any point on the beam, the measurement was repeated to ensure the accuracy and completeness of the data. The experiment was carried out in a spacious indoor space with stable natural lighting to minimize external interference. All children were required to wear tight-fitting sportswear to ensure the accuracy of data collection and non-slip athletics shoes to ensure safety.

### 2.4 Data processing

Upon completion of the walking capture, the recorded videos were automatically uploaded to OpenCap's web application. The built-in algorithm code automatically computed the three-dimensional marker positions and joint kinematics and output them in an OpenSim file format. Subsequently, based on muscle-driven simulation of joint kinematics, the kinetic parameters were estimated using the OpenCap processing library. The accuracy of OpenCap's kinematic and kinetic estimates has been validated against gold standard marker-based motion capture and force plates (22). The processing environment included Python (version 3.8, Python Software Foundation) and OpenSim (version 4.5, Stanford University, USA).

To minimize the influence of random variability, three consecutive gait cycles were selected. For the comparison of continuous time-series variables, the reaction forces at the hip, knee, and ankle joints were normalized as percentages of the gait cycle (101 data points, ranging from 0% to 100%). The gait cycle was defined as the interval between the heel-strike of a given foot and its subsequent heel-strike (25). Data were filtered using a second-order low-pass Butterworth filter with a cutoff frequency of 6 Hz (26). Both normalization and filtering processes were performed using Python.

### 2.5 Statistical analyses

This study analyzed one-dimensional time series of joint reaction force throughout the gait cycle. We employed Python (v3.8, Python Software Foundation) software for data statistical tests. The code utilized one-dimensional statistical parametric mapping (spm1d) developed by Pataky (27) based on random field theory. Shapiro–Wilk test within the script was performed to assess the normality of the data distribution. Paired *t*-test with a non-parametric approach (SnPM) were performed to analyze and compare hip, knee, and ankle joint reaction force between OGW and BBW. Data visualization and plotting were performed using Origin software (v 2024, OriginLab Corporation, Inc., Northampton, MA). The statistical significance was set at 0.05.

## 3 Results

**Figure 2:**
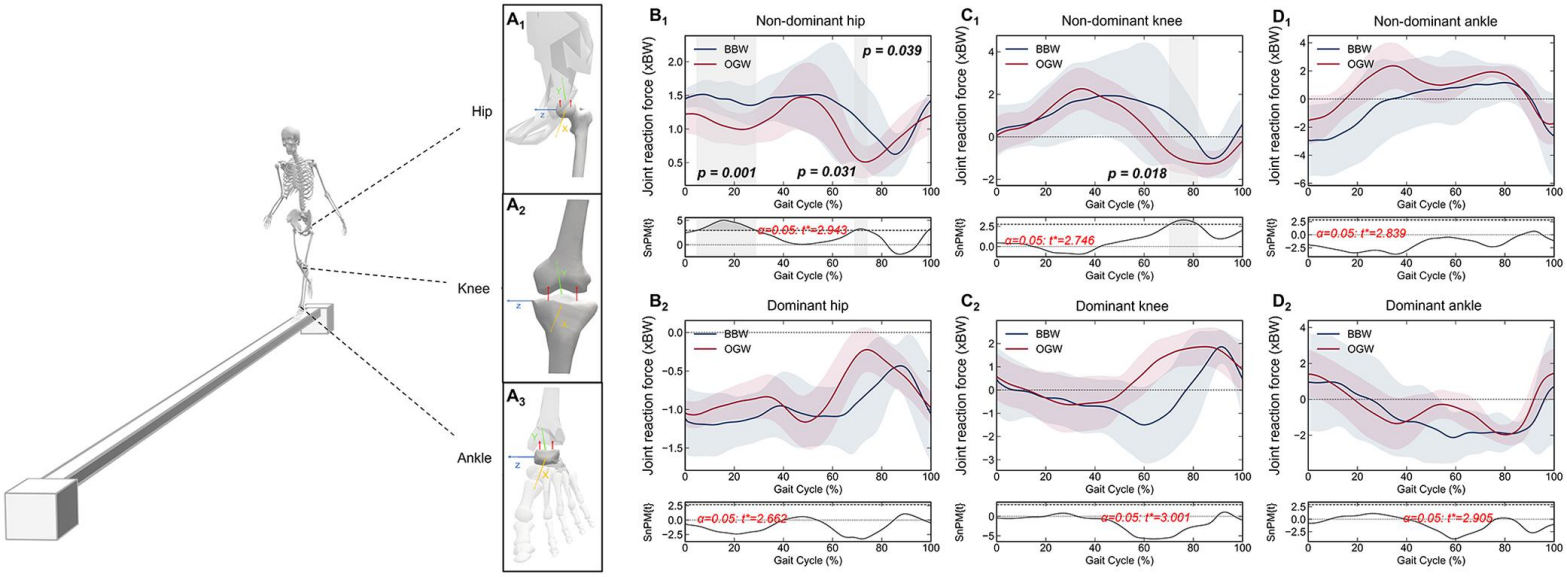
Schematic diagram of balance beam walking and hip **(A_1_)**, knee **(A_2_)**, and ankle **(A_3_)** medial-lateral reaction force. One-dimensional time series curves of joint reaction force on the non-dominant and dominant hip **(B_1_,B_2_)**, knee **(C_1_,C_2_)** and ankle (**D_1_,D_2_)** joint during the gait cycle under the balance beam walking (BBW) and overground walking (OGW). The shaded gray bar areas indicate the specific phase differences of the gait cycle. The *p*-value indicates statistical significance.

Schematic diagrams of the medial and lateral reaction forces at the hip, knee, and ankle joints are shown in Figure 2A_1_–A_3_. Under the BBW condition, the non-dominant hip joint (Figure 2B_1_) exhibited greater medial reaction forces from loading response to mid-stance at 4.82–28.72% (*p* = 0.001), from initial swing to mid-swing at 68.99–74.11% (*p* = 0.031), and during the terminal swing phase at 98.91–100.00% (*p* = 0.039), whereas the dominant hip joint (Figure 2B_2_) showed no significant differences. Compared with the dominant knee, the non-dominant knee joint (Figure 2C_1_**)** exhibited lower lateral reaction forces during the transition phase from initial swing to mid-swing at 70.34–81.81% of the gait cycle (*p* = 0.018). Under the OGW condition, the non-dominant knee joint exhibited a negative reaction force at 70.34% of the gait cycle, indicating that the reaction force acted on the lateral side. However, under the BBW condition, the knee reaction force exhibited a slower transition from the medial side to the lateral side at 79% of the gait cycle. Under both conditions, no significant differences in reaction forces were observed at the dominant knee joint (Figure 2C_2_) or ankle joint (Figure 2D_1_,D_2_).

## 4 Discussion

By comparing the joint reaction forces in the frontal plane under BBW and OGW conditions, we found that 4-year-old children exhibited a unilateral loading shift strategy during dynamic balance control tasks. Children enhanced control of lateral body stability by increasing reaction forces in the mediolateral direction of the non-dominant lower limb (especially the hip and knee joints), thereby maintaining balance. Such adjustments were mainly reflected in the regulation of unilateral lower limb loading rather than symmetric regulation of bilateral loading, indicating a compensatory gait pattern characterized by unilateral regulation and contralateral coordination.

BBW is a functional task that requires highly precise control and regulation of balance and is commonly used to assess motor coordination and stability (28, 29). Compared to normal walking, unstable gait tends to cause the trunk to sway laterally (30), which is negatively correlated with age (31). This instability is particularly pronounced in tasks requiring balance adjustments (32). Previous studies have shown that gait stability can be maintained across different movement patterns through the coordinated action of the hip, knee, and ankle joints along with their muscle synergies (33).

As the primary connection between the trunk and lower limbs, the hip joint serves to generate and transmit force (34). When walking conditions change, gait patterns consequently alter (35, 36). Previous studies have shown that a wider step helps improve lateral stability (37), while a narrower step width increases hip joint reaction forces (38). In the current study, the non-dominant hip joint exhibited greater reaction forces at the early and late swing phases under the BBW condition, which is consistent with the findings of previous studies (38, 39). The reaction force of the non-dominant knee was negative under OGW, indicating that the knee joint was subjected to a force in the medial direction, whereas the reaction force of the left knee gradually transitioned from the lateral to the medial side under BBW, exhibiting a slower transition pattern. Previous studies have found that changes in gait patterns lead to shifts in joint loading patterns (40–43), such as a slower transition from the stance to the swing phase and a significant increase of double support time, thereby reducing joint pressure and avoiding rapid loading impacts to prevent joint injuries (44, 45). The knee joint is primarily responsible for absorbing impact and transmitting ground reaction forces, playing a critical role in overall balance adjustment and fine-tuning of gait during dynamic balance tasks (1, 46). Compared to the hip and ankle joints, the knee joint has a smaller range of motion in the frontal plane and therefore has limited direct control over balance. Its main function is to assist in maintaining postural control (47). Gait adjustment can reduce knee varus moment and medial reaction force, but with little effect on the overall reaction force (48, 49).

The reaction forces at the dominant hip and knee joint did not vary significantly under OGW and BBW conditions. Typically, healthy preschoolers exhibit symmetrical gait patterns when walking normally. However, changes in walking conditions lead to increased unilateral limb loading, resulting in significant gait asymmetry (50, 51). Subsequently, this asymmetry affects the symmetry of the hip joint reaction forces (52). Changes in gait patterns may lead to variability in hip joint loading peaks and the occurrence (38, 39, 53), thereby forming continuous, unstructured adaptive patterns rather than abrupt changes (52, 54). This decentralized loading regulation strategy may reflect that preschool children have not yet established mature motor control patterns in functional tasks of higher difficulty, as evidenced by the dominant hip joint assuming a higher functional role in synergistic regulation. Studies have shown that the dominant leg exhibits more pronounced regulatory functions, while the non-dominant leg contributes more to support assistance, resulting in a unilateral regulation and contralateral assistance gait pattern (55).

Joint loading is influenced by joint kinematics, muscle activation, and neuromuscular control. The greater the joint loading, the higher the gait variability (56, 57). Variations in joint loading appear to be induced by gait variability, but from a control theory perspective, this variability actually reflects the presence of motor redundancy (58). When attempting to perform a motor task, children regulate through various muscle combinations and movement patterns to achieve the same motor goal (59). By increasing antagonist muscle activity, dynamic knee joint stiffness is improved, thereby overcoming knee joint instability (60). Typically, ankle control strategies serve as the primary mechanism for adults to counteract environmental disturbances (61, 62). However, preschoolers exhibit weaker ankle dorsiflexion and inversion-eversion regulatory capabilities, along with differences in muscle activation patterns (63). When faced with unstable conditions, children's ankle joints cannot perform fine-tuning adjustments as effectively as adults (64). Moderate motor variability is not a sign of control failure but rather reflects children's continuous adjustments and optimization of movement patterns in response to environmental conditions. However, long-term irregular distribution of loading on the lower limbs may cause excessive pressure on joints, impairing their ability to absorb impact and ultimately resulting in joint injury, such as osteoarthritis and fractures (65, 66).

The core challenge of beam walking lies in maintaining lateral balance on a narrow support surface (28). Lateral stability is crucial for dynamic balance regulation in gait (67), and the coordination of the hip, knee, and ankle joints and associated muscles significantly influences the adjustment of the center of gravity in the frontal plane (68). The primary mechanism for adults to counteract interference conditions is ankle control strategies, whereas this study found that 4-year-old children seem to prefer hip control strategies for beam walking. This has important implications for understanding the overall balance control mechanisms and injury prevention in preschoolers.

## 5 Limitation

Assessing functional gait tasks through joint reaction forces enables a more comprehensive understanding of preschool children's balance control strategies from a biomechanical perspective. As a functional training tool, beam walking places emphasis on continuity and prevention of excessive lateral sway. This facilitates targeted rehabilitation training for preschool children with impaired gait function. Cognition and attention may influence balance performance (69), which was not considered in the present study, representing a limitation that warrants attention. Cognitive processes related to balance in children vary across tasks, influenced by perceptual characteristics and task specificity. When children focus on task completion, these processes alter gait patterns, thereby increasing gait variability. Children with superior balance abilities tend to perform tasks more efficiently (69–71). However, this relationship requires further confirmation in preschool children. Integrating electroencephalography or eye-tracking technology could analyze the neural mechanisms linking attentional allocation with gait variability in depth (69, 72). This may provide a more comprehensive neurocentral perspective for investigating functional gait task control strategies in preschool children.

## 6 Conclusion

Beam walking in 4-year-old children significantly increased the demand for loading regulation in the non-dominant lower limb joints, and the motor control strategies demonstrated pronounced asymmetry. In functional tasks, preschoolers require greater joint loading regulation capabilities to maintain body stability. Children's neuromuscular control system is still developing at this age, but they have already acquired a certain degree of adaptability.

## Data Availability

The raw data supporting the conclusions of this article will be made available by the authors, without undue reservation.

## References

[1] Cheng KB. Does knee motion contribute to feet-in-place balance recovery? J Biomech. (2016) 49:1873–80. 10.1016/j.jbiomech.2016.04.026 27155745

[2] Larsen LR Kristensen PL Junge T Møller SF Juul-Kristensen B Wedderkopp N. Motor performance as risk factor for lower extremity injuries in children. Med Sci Sports Exerc. (2016) 48:1136–43. 10.1249/MSS.0000000000000877 26765628

[3] Sutherland DH Olshen R Cooper L Woo SL. The development of mature gait. J Bone Joint Surg Am. (1980) 62:336–53. 10.2106/00004623-198062030-00004 7364807

[4] Steindl R Kunz K Schrott-Fischer A Scholtz AW. Effect of age and sex on maturation of sensory systems and balance control. Dev Med Child Neurol. (2006) 48:477–82. 10.1111/j.1469-8749.2006.tb01299.x 16700940

[5] Verbecque E Vereeck L Van de Heyning P Hallemans A. Gait and its components in typically developing preschoolers. Gait Posture. (2017) 58:300–6. 10.1016/j.gaitpost.2017.08.012 28843930

[6] Sawers A Hafner B. Narrowing beam-walking is a clinically feasible approach for assessing balance ability in lower-limb prosthesis users. J Rehabil Med. (2018) 50:457–64. 10.2340/16501977-2329 29616279 PMC6171346

[7] Symeonidou ER Esposito NM Reyes RD Ferris DP. Practice walking on a treadmill-mounted balance beam modifies beam walking sacral movement and alters performance in other balance tasks. PLoS One. (2023) 18:1–15. 10.1371/journal.pone.0283310 PMC10270570 37319297

[8] Symeonidou E-R Ferris DP. Intermittent visual occlusions increase balance training effectiveness. Front Hum Neurosci. (2022) 16:1–6. 10.3389/fnhum.2022.748930 PMC9083907 35547194

[9] Giacalone WR Rarick GL. Dynamic balance of preschool children as reflected by performance on beam-walking tasks. J Genet Psychol. (1985) 146:307–18. 10.1080/00221325.1985.9914460

[10] Maes L De Kegel A Van Waelvelde H Dhooge I. Association between vestibular function and motor performance in hearing-impaired children. Otol Neurotol. (2014) 35:e343–7. 10.1097/MAO.0000000000000597 25275872

[11] Möhring W Klupp S Zumbrunnen R Segerer R Schaefer S Grob A. Age-related changes in children’s cognitive–motor dual tasking: evidence from a large cross-sectional sample. J Exp Child Psychol. (2021) 206:105103. 10.1016/j.jecp.2021.105103 33639577

[12] Waterval NFJ Brehm M-A Ploeger HE Nollet F Harlaar J. Compensations in lower limb joint work during walking in response to unilateral calf muscle weakness. Gait Posture. (2018) 66:38–44. 10.1016/j.gaitpost.2018.08.016 30145473

[13] Paez-Moguer J Montes-Alguacil J Garcia-Paya I Medina-Alcantara M Evans AM Gijon-Nogueron G. Variation of spatiotemporal parameters in school children carrying different backpack loads: a cross sectional study. Sci Rep. (2019) 9:12192. 10.1038/s41598-019-48675-3 31434980 PMC6704062

[14] Kimoto M Okada K Mitobe K Saito M Sakamoto H. Strategies for unplanned gait termination at comfortable and fast walking speeds in children with cerebral palsy. J Biomech. (2024) 176:112349. 10.1016/j.jbiomech.2024.112349 39366271

[15] Cimolin V Cau N Sartorio A Capodaglio P Galli M Tringali G Symmetry of gait in underweight, normal and overweight children and adolescents. Sensors. (2019) 19:2054. 10.3390/s19092054 31052569 PMC6539288

[16] Donelan JM Shipman DW Kram R Kuo AD. Mechanical and metabolic requirements for active lateral stabilization in human walking. J Biomech. (2004) 37:827–35. 10.1016/j.jbiomech.2003.06.002 15111070

[17] Guan Y Bredin S Taunton J Jiang Q Wu L Kaufman K Bilateral difference between lower limbs in children practicing laterally dominant vs. Non-laterally dominant sports. Eur J Sport Sci. (2021) 21:1092–100. 10.1080/17461391.2020.1814425 32835613

[18] Faul F Erdfelder E Lang A-G Buchner A. G*power 3: a flexible statistical power analysis program for the social, behavioral, and biomedical sciences. Behav Res Methods. (2007) 39:175–91. 10.3758/BF03193146 17695343

[19] Ren X Lutter C Kebbach M Bruhn S Bader R Tischer T. Lower extremity joint compensatory effects during the first recovery step following slipping and stumbling perturbations in young and older subjects. BMC Geriatr. (2022) 22:656. 10.1186/s12877-022-03354-3 35948887 PMC9367084

[20] Brown D Ahn J Simpkins C Yang F. Dominant or nondominant leg, which one is used to recover balance after a simulated slip during standing? Gait Post. (2025) 121:19–24. 10.1016/j.gaitpost.2025.04.028 40305965

[21] Ke D Lu D Cai G Wang X Zhang J Suzuki K. Chronological and skeletal age in relation to physical fitness performance in preschool children. Front Pediatr. (2021) 9:1–8. 10.3389/fped.2021.641353 PMC8160222 34055684

[22] Uhlrich SD Falisse A Kidziński Ł Muccini J Ko M Chaudhari AS Opencap: human movement dynamics from smartphone videos. PLoS Comput Biol. (2023) 19:1–26. 10.1371/journal.pcbi.1011462 PMC10586693 37856442

[23] Latorre-Román PA Martínez-Redondo M Párraga-Montilla JA Lucena-Zurita M Manjón-Pozas D González PJC Analysis of dynamic balance in preschool children through the balance beam test: a cross-sectional study providing reference values. Gait Posture. (2021) 83:294–9. 10.1016/j.gaitpost.2020.11.004 33246259

[24] Li R Liu M Zhu J Li R Zhao H Zhang L. Age and gender differences in static and dynamic balance of Chinese preschool children. Front Physiol. (2022) 13:1–10. 10.3389/fphys.2022.1013171 PMC9618940 36324303

[25] Al-Shuka HFN Rahman MH Leonhardt S Ciobanu I Berteanu M. Biomechanics, actuation, and multi-level control strategies of power-augmentation lower extremity exoskeletons: an overview. Int J Dynamic Control. (2019) 7:1462–88. 10.1007/s40435-019-00517-w

[26] Ren X Lutter C Kebbach M Bruhn S Yang Q Bader R Compensatory responses during slip-induced perturbation in patients with knee osteoarthritis compared with healthy older adults: an increased risk of falls? Front Bioeng Biotechnol. (2022) 10:1–13. 10.3389/fbioe.2022.893840 PMC9240265 35782515

[27] Pataky TC. One-dimensional statistical parametric mapping in python. Comput Methods Biomech Biomed Engin. (2012) 15:295–301. 10.1080/10255842.2010.527837 21756121

[28] Sawers A Ting LH. Beam walking can detect differences in walking balance proficiency across a range of sensorimotor abilities. Gait Posture. (2015) 41:619–23. 10.1016/j.gaitpost.2015.01.007 25648493

[29] Hortobágyi T Vetrovsky T Uematsu A Sanders L da Silva Costa AA Batistela RA Walking on a balance beam as a new measure of dynamic balance to predict falls in older adults and patients with neurological conditions. Sports Med Open. (2024) 10:59. 10.1186/s40798-024-00723-7 38775922 PMC11111647

[30] Van de Warrenburg BPC Bakker M Kremer BPH Bloem BR Allum JHJ. Trunk sway in patients with spinocerebellar ataxia. Mov Disord. (2005) 20:1006–13. 10.1002/mds.20486 15838852

[31] Gill J Allum JHJ Carpenter MG Held-Ziolkowska M Adkin AL Honegger F Trunk sway measures of postural stability during clinical balance tests: effects of age. J Gerontol A Biol Sci Med Sci. (2001) 56:M438–47. 10.1093/gerona/56.7.M438 11445603

[32] Zhang F Liu P Ou Y Huang Q Song R Dou Z Trunk and head control during walking in patients with unilateral vestibular hypofunction: effect of lower limb somatosensory input. Am J Phys Med Rehabil. (2019) 98:906–13. 10.1097/PHM.0000000000001223 31116108

[33] Liu YX Gutierrez-Farewik EM. Joint kinematics, kinetics and muscle synergy patterns during transitions between locomotion modes. IEEE Trans Biomed Engin. (2023) 70:1062–71. 10.1109/TBME.2022.3208381 36129869

[34] Polkowski GG Clohisy JC. Hip biomechanics. Sports Med Arthrosc. (2010) 18:56–62. 10.1097/JSA.0b013e3181dc5774 20473123

[35] Suri A VanSwearingen J Rosano C Brach JS Redfern MS Sejdić E Uneven surface and cognitive dual-task independently affect gait quality in older adults. Gait and Posture. (2023) 106:34–41. 10.1016/j.gaitpost.2023.08.010 37647710 PMC10591986

[36] Dussault-Picard C Cherni Y Dixon PC. Spatiotemporal characteristics of gait when walking on an uneven surface in children with cerebral palsy. Sci Rep. (2025) 15:4912. 10.1038/s41598-025-89280-x 39929957 PMC11811155

[37] Vistamehr A Neptune RR. Differences in balance control between healthy younger and older adults during steady-state walking. J Biomech. (2021) 128:110717. 10.1016/j.jbiomech.2021.110717 34530294

[38] Wesseling M de Groote F Meyer C Corten K Simon J-P Desloovere K Gait alterations to effectively reduce hip contact forces. J Orthop Res. (2015) 33:1094–102. 10.1002/jor.22852 25676535

[39] Shepherd MC Gaffney BMM Song K Clohisy JC Nepple JJ Harris MD. Femoral version deformities alter joint reaction forces in dysplastic hips during gait. J Biomech. (2022) 135:1–16. 10.1016/j.jbiomech.2022.111023 PMC9064981 35247684

[40] Shelburne KB Torry MR Pandy MG. Muscle, ligament, and joint-contact forces at the knee during walking. Med Sci Sports Exercise. (2005) 37:1948–56. 10.1249/01.mss.0000180404.86078.ff 16286866

[41] Thoma LM McNally MP Chaudhari AM Best TM Flanigan DC Siston RA Differential knee joint loading patterns during gait for individuals with tibiofemoral and patellofemoral articular cartilage defects in the knee. Osteoarthritis Cartilage. (2017) 25:1046–54. 10.1016/j.joca.2017.02.794 28232097

[42] Sharifi M Shirazi-Adl A Marouane H. Sensitivity of the knee joint response, muscle forces and stability to variations in gait kinematics-kinetics. J Biomech. (2020) 99:109472. 10.1016/j.jbiomech.2019.109472 31708244

[43] Costello KE Felson DT Neogi T Segal NA Lewis CE Gross KD Ground reaction force patterns in knees with and without radiographic osteoarthritis and pain: descriptive analyses of a large cohort (the multicenter osteoarthritis study). Osteoarthritis Cartilage. (2021) 29:1138–46. 10.1016/j.joca.2021.03.009 33757856 PMC8319033

[44] Chen CPC Chen MJL Pei Y-C Lew HL Wong P-Y Tang SFT. Sagittal plane loading response during gait in different age groups and in people with knee osteoarthritis. Am J Phys Med Rehabi. (2003) 82:307–12. 10.1097/01.phm.0000056987.33630.56 12649658

[45] Valente G Grenno G Dal Fabbro G Zaffagnini S Taddei F. Medial and lateral knee contact forces during walking, stair ascent and stair descent are more affected by contact locations than tibiofemoral alignment in knee osteoarthritis patients with varus malalignment. Front Bioeng Biotechnol. (2023) 11:1–10. 10.3389/fbioe.2023.1254661 PMC10507691 37731759

[46] Wan G Wang P Han Y Liang J. Torque modulation mechanism of the knee joint during balance recovery. Comput Biol Med. (2024) 175:108492. 10.1016/j.compbiomed.2024.108492 38678940

[47] Iqbal K Pai Y-C. Predicted region of stability for balance recovery:motion at the knee joint can improve termination of forward movement. J Biomech. (2000) 33:1619–27. 10.1016/S0021-9290(00)00129-9 11006386

[48] Richards RE Andersen MS Harlaar J van den Noort JC. Relationship between knee joint contact forces and external knee joint moments in patients with medial knee osteoarthritis: effects of gait modifications. Osteoarthritis Cartilage. (2018) 26:1203–14. 10.1016/j.joca.2018.04.011 29715509

[49] Starkey SC Lenton GK Saxby DJ Hinman RS Bennell KL Wrigley T Effect of exercise on knee joint contact forces in people following medial partial meniscectomy: a secondary analysis of a randomised controlled trial. Gait Posture. (2020) 79:203–9. 10.1016/j.gaitpost.2020.04.025 32438267

[50] Liikavainio T Isolehto J Helminen HJ Perttunen J Lepola V Kiviranta I Loading and gait symmetry during level and stair walking in asymptomatic subjects with knee osteoarthritis: importance of quadriceps femoris in reducing impact force during heel strike? Knee. (2007) 14:231–8. 10.1016/j.knee.2007.03.001 17451958

[51] Christensen JC LaStayo PC Mizner RL Marcus RL Pelt CE Stoddard GJ Joint mechanical asymmetries during low- and high-demand mobility tasks: comparison between total knee arthroplasty and healthy-matched peers. Gait Post. (2018) 60:104–10. 10.1016/j.gaitpost.2017.11.017 29175639

[52] Koller W Baca A Kainz H. The gait pattern and not the femoral morphology is the main contributor to asymmetric hip joint loading. PLoS One. (2023) 18:e0291789. 10.1371/journal.pone.0291789 37751435 PMC10522038

[53] Giarmatzis G Jonkers I Wesseling M Van Rossom S Verschueren S. Loading of hip measured by hip contact forces at different speeds of walking and running. J Bone Miner Res. (2015) 30:1431–40. 10.1002/jbmr.2483 25704538

[54] Bergmann G Bergmann G Deuretzabacher G Deuretzabacher G Heller M Heller M Hip forces and gait patterns from rountine activities. J Biomech. (2001) 34:859–71. 10.1016/S0021-9290(01)00040-9 11410170

[55] Seeley MK Umberger BR Shapiro R. A test of the functional asymmetry hypothesis in walking. Gait Posture. (2008) 28:24–8. 10.1016/j.gaitpost.2007.09.006 17997095

[56] Hubley-Kozey CL Deluzio KJ Landry SC McNutt JS Stanish WD. Neuromuscular alterations during walking in persons with moderate knee osteoarthritis. J Electromyogr Kinesiol. (2006) 16:365–78. 10.1016/j.jelekin.2005.07.014 16213159

[57] Bailey CA Porta M Pilloni G Arippa F Côté JN Pau M. Does variability in motor output at individual joints predict stride time variability in gait? Influences of age, sex, and plane of motion. J Biomech. (2020) 99:109574. 10.1016/j.jbiomech.2019.109574 31870659

[58] Dingwell JB John J Cusumano JP. Do humans optimally exploit redundancy to control step variability in walking? PLoS Comput Biol. (2010) 6:14. 10.1371/journal.pcbi.1000856 PMC2904769 20657664

[59] Waugh CM Korff T Blazevich AJ. Developmental differences in dynamic muscle-tendon behaviour: implications for movement efficiency. J Exp Biol. (2017) 220:1287–94. 10.1242/jeb.127951 28108669

[60] Zeni JA Higginson JS. Dynamic knee joint stiffness in subjects with a progressive increase in severity of knee osteoarthritis. Clin Biomech. (2009) 24:366–71. 10.1016/j.clinbiomech.2009.01.005 PMC2696188 19250725

[61] van den Bogaart M Bruijn SM van Dieën JH Meyns P. The use of the ankle strategy to restore balance during perturbed walking. Gait Posture. (2018) 65:320–1. 10.1016/j.gaitpost.2018.06.206

[62] Kaminishi K Chiba R Takakusaki K Ota J. Increase in muscle tone promotes the use of ankle strategies during perturbed stance. Gait Posture. (2021) 90:67–72. 10.1016/j.gaitpost.2021.08.003 34411975

[63] Kurz E Faude O Roth R Zahner L Donath L. Ankle muscle activity modulation during single-leg stance differs between children, young adults and seniors. Eur J Appl Physiol. (2018) 118:239–47. 10.1007/s00421-017-3764-0 29188450

[64] Fox EJ Moon H Kwon MH Chen YT Christou EA. Neuromuscular control of goal-directed ankle movements differs for healthy children and adults. Eur J Appl Physiol. (2014) 114:1889–99. 10.1007/s00421-014-2915-9 24906445

[65] Buckwalter JA Anderson DD Brown TD Tochigi Y Martin JA. The roles of mechanical stresses in the pathogenesis of osteoarthritis. Cartilage. (2013) 4:286–94. 10.1177/1947603513495889 25067995 PMC4109888

[66] Robinson PG Campbell VB Murray AD Nicol A Robson J. Stress fractures: diagnosis and management in the primary care setting. Br J Gen Pract. (2019) 69:209–300. 10.3399/bjgp19X702137 30923162 PMC6428476

[67] Sidaway B Bennett J Berenyi S Bryant P Cushman K DiMonte R The identification of fall risk through tests of mediolateral stability during gait. Exp Gerontol. (2022) 163:111803. 10.1016/j.exger.2022.111803 35413409

[68] Pandy MG Lin YC Kim HJ. Muscle coordination of mediolateral balance in normal walking. J Biomech. (2010) 43:2055–64. 10.1016/j.jbiomech.2010.04.010 20451911

[69] Weedon BD Esser P Collett J Izadi H Inacio M Joshi S The effects of cognitive-motor interference on walking performance in adolescents with low balance. Gait Pos. (2024) 114:202–7. 10.1016/j.gaitpost.2024.09.016 39357116

[70] Messer DJ Pine KJ Butler C. Children’s behaviour and cognitions across different balance tasks. Learn Instr. (2008) 18:42–53. 10.1016/j.learninstruc.2006.09.008

[71] O’Hagan AD Behan S Peers C Belton S O’Connor N Issartel J. Do our movement skills impact our cognitive skills? Exploring the relationship between cognitive function and fundamental movement skills in primary school children. J Sci Med Sport. (2022) 25:871–7. 10.1016/j.jsams.2022.08.001 36064502

[72] Reiser JE Wascher E Arnau S. Recording mobile EEG in an outdoor environment reveals cognitive-motor interference dependent on movement complexity. Sci Rep. (2019) 9:1–14. 10.1038/s41598-019-49503-4 31511571 PMC6739372

